# Molecular Crosstalk between the Hepatitis C Virus and the Extracellular Matrix in Liver Fibrogenesis and Early Carcinogenesis

**DOI:** 10.3390/cancers13092270

**Published:** 2021-05-09

**Authors:** Emma Reungoat, Boyan Grigorov, Fabien Zoulim, Eve-Isabelle Pécheur

**Affiliations:** Université Lyon, University Claude Bernard, CNRS 5286, Inserm U1052, Centre de Recherche en Cancérologie de Lyon (CRCL), 69008 Lyon, France; reungoatemma@gmail.com (E.R.); boyan.grigorov@inserm.fr (B.G.); fabien.zoulim@inserm.fr (F.Z.)

**Keywords:** liver fibrosis, cirrhosis, chronic hepatitis C, carcinogenesis, extracellular matrix

## Abstract

**Simple Summary:**

In the era of direct-acting antivirals against the hepatitis C virus (HCV), curing chronic hepatitis C has become a reality. However, while replicating chronically, HCV creates a peculiar state of inflammation and oxidative stress in the infected liver, which fuels DNA damage at the onset of HCV-induced hepatocellular carcinoma (HCC). This cancer, the second leading cause of death by cancer, remains of bad prognosis when diagnosed. This review aims to decipher how HCV durably alters elements of the extracellular matrix that compose the liver microenvironment, directly through its viral proteins or indirectly through the induction of cytokine secretion, thereby leading to liver fibrosis, cirrhosis, and, ultimately, HCC.

**Abstract:**

Chronic infection by the hepatitis C virus (HCV) is a major cause of liver diseases, predisposing to fibrosis and hepatocellular carcinoma. Liver fibrosis is characterized by an overly abundant accumulation of components of the hepatic extracellular matrix, such as collagen and elastin, with consequences on the properties of this microenvironment and cancer initiation and growth. This review will provide an update on mechanistic concepts of HCV-related liver fibrosis/cirrhosis and early stages of carcinogenesis, with a dissection of the molecular details of the crosstalk during disease progression between hepatocytes, the extracellular matrix, and hepatic stellate cells.

## 1. Introduction

A recent report from the International Agency for Research on Cancer states that 15% of new cancer cases in 2012 were attributable to carcinogenic infections [1], caused by oncogenic viruses: human papillomavirus for cervical carcinoma, Epstein–Barr virus for Burkitt’s and Hodgkin’s lymphomas and nasopharynx carcinoma, and hepatitis B and C viruses (HBV and HCV, respectively) for hepatocellular carcinoma (HCC). Of note, 73% of HCC cases are attributable to HBV and HCV [1]. It is the fifth most common cancer worldwide and the second leading cause of cancer death. Its prognosis is poor, with a 5-year survival of only 18% [2]; moreover, diagnosis often occurs late, and curative therapy is not available. Over decades, chronic inflammation and oxidative stress induced by causative agents lead to chronic hepatic injury, with excessive wound healing and deposition of connective tissue (fibrosis), and disruption of hepatic architecture and function, with proliferation of regenerating hepatocytes (cirrhosis), eventually leading to chromosomal aberrations and malignant transformation of proliferating hepatocytes (HCC) [3]. However, HBV and HCV exhibit different pathogenesis and carcinogenic properties, recapitulated in Table 1. HCV displays a predominantly cytoplasmic life cycle, which renders it more likely to drive carcinogenesis through the alteration of cell signaling and metabolism and modulation of immune responses. These processes fuel chronic inflammation, oxidative stress, and repair mechanisms underlying liver fibrosis, cirrhosis, and HCC. Nevertheless, some HCV proteins have been shown to alter cell cycle checkpoint machinery such as the retinoblastoma protein Rb and the mitotic spindle [4]. With the discovery of direct-acting antivirals, HCV can be eliminated, and 95 to 99% of chronically infected patients can be considered cured [5]. Favorable outcomes of infection are now also obtained for cirrhotic and cancer patients.

Approximately 85% of HCV-infected individuals develop chronic hepatitis C. At present, ≈80 million individuals are chronically infected worldwide [1]. Patients with chronic hepatitis C are at risk of increased fibrosis progression, with subsequent complications of cirrhosis and HCC [6,7]. Based on the natural history of chronic hepatitis C, at least 30% will develop liver fibrosis, 7–18% will develop cirrhosis, and 1–5% HCC within 20–30 years [8]. A projection of the World Health Organization estimates that more than 1 million patients will die from liver cancer in 2030 [2].

After years of interferon-based therapies, the introduction of new antivirals directly targeting HCV replication (direct-acting antivirals (DAAs)) and achieving sustained virological response (SVR) in more than 95% of treated patients raised great hopes of a marked reduction in HCC occurrence and recurrence in patients with a history of previous liver cancer treated surgically. However, recent clinical observations report somewhat conflicting data [9]. SVR induced by anti-HCV therapies based upon interferon or upon DAAs may result in distinct post-SVR HCC risk [10]. HCV may lead to irreversible changes in cellular signaling (epigenetic events [11], imprinting), and recent data tend to indicate that chronic hepatitis C durably disrupts the balance of inflammatory mediators, even after HCV clearance [12]. These features could underlie a residual risk of carcinogenesis after viral clearance [11]. For example, a variant in the core protein of HCV genotype 1b is associated with increased HCC incidence post SVR [13]. A more recent study pointed at sustained oncogenic transcriptomic profiles in liver tissues after HCV eradication with DAAs [14]. Among them, increased serum levels of CYR61 could be a possible biomarker of HCC post-SVR [14] (see Section 3.2.4). Thus, SVR is a virological cure but is not necessarily translated into a cure from risks of liver disease, particularly for patients with cirrhotic-stage fibrosis.

Contamination with HCV occurs from a breach through the blood circulation, from which the virus is transported to the liver, where its target cells are hepatocytes. Virions are composed of three structural proteins: the capsid or core protein, which compacts the viral genome, and the envelope glycoproteins E1 and E2, which permit viral entry through recognition of several surface receptors. HCV replication, involving nonstructural viral proteins (NS2, 3, 4A, 4B, 5A, 5B) is restricted to the cytoplasm of infected cells, where it engages the endoplasmic reticulum (ER) and lipid droplets. Unlike other carcinogenic viruses, HCV entirely replicates outside the nucleus of hepatocytes, and no latency or persistence factor is synthesized during its life cycle [15]. The deleterious effects of chronic hepatitis C are therefore anticipated to occur through a subtle interplay between viral determinants and the liver microenvironment in which the virus propagates. Studying HCV pathogenesis thus implies the thorough study of cellular and tissular alterations induced throughout chronic infection.

Here, we will focus on chronic hepatitis C-related fibrogenesis and early carcinogenesis and examine the actors of these phenomena and their entanglement. Importantly, knowledge gained from HCV could be useful for other etiologies of chronic liver diseases, viral or not, as some actors are common to various etiologies. For such purposes, molecular details of the crosstalk between some liver cells and the hepatic extracellular matrix (ECM) will be dissected.

## 2. Main Actors of Liver Fibrosis

In the human liver, 80% of cells are hepatocytes. These epithelial cells are derived from the bipotential progenitor cells called hepatoblasts or hepatic progenitor cells (HPCs), also capable of differentiation toward cholangiocytes or bile duct cells, which delineate bile canaliculi [16]. Other cell types are hepatic stellate cells (HSCs), acting as reservoirs of vitamin A; endothelial cells, forming liver capillary sinusoids; and liver-resident macrophages (Kupffer cells). These macrophages are cells of great plasticity. Liver injury triggers their activation, leading to inflammatory cytokine and chemokine release, which fuels inflammation and fibrogenesis. However, if liver injury ceases, they switch their phenotype toward reparative phagocytes under specific signals, thereby promoting tissue repair and regression of fibrosis [17]. We will nevertheless only focus on hepatocytes and HSCs, as these cells produce components of the hepatic ECM, and hepatocytes are the targets of HCV infection. Nutrients, molecules from the hepatic microenvironment, and substances to be transported to the bile arrive from sinusoid capillaries, forming a fenestrated endothelium. The region between blood capillary sinusoids and hepatocytes is the space of Disse, containing HSCs and filled by the hepatic ECM. This space, part of the liver connective tissue [18], is an active zone of exchange between blood and hepatocytes.

In normal liver, HSCs are quiescent and exhibit a spindle-like shape; their most characteristic feature is the storage of retinoids in intracellular droplets [19]. HSCs also play a major role in liver development and regeneration by expressing and secreting hepatocyte mitogens such as hepatocyte growth factor (HGF) or epidermal growth factor (EGF) [15]. In injured liver, HSCs become activated, with a continuum of changes in gene expression during activation. Activated HSCs migrate and accumulate at the sites of tissue repair, secreting large amounts of ECM, mainly type I collagen fibrils that cross-link and deposit in the space of Disse, matrix metalloproteases (MMPs), and their inhibitors tissue inhibitors of MMPs (TIMPs). This contributes to the regulation of ECM remodeling. They also differentiate into myofibroblast-like cells expressing α-smooth muscle actin (α-SMA) [20]. Loss of retinoids and lipid droplets is concomitant to a de novo expression of receptors of profibrotic and chemotactic factors.

The hepatic ECM, as other ECM, can be subdivided into interstitial matrix and basement membrane. Liver ECM forms a very limited compartment of low density within the normal liver [21], comprising less than 3% of the relative area on a tissue section and approximately 0.5% of the wet weight [22]. It is of major importance in liver physiology through its scaffolding effect and roles in biological functions such as cell proliferation, migration, and differentiation. Liver ECM proteins are mostly detected in the Glisson capsule (the connective tissue surrounding the liver), portal tracks, central veins, and in the subendothelial space of Disse. Collagens, fibronectin, laminins, proteoglycans, and matricellular proteins (such as thrombospondins, tenascins, and osteopontin) are the main ECM components in the normal liver. Fibrillar collagens type I, III, and V are mostly interstitial, in the portal and central regions. They are produced by activated HSCs and emanate from the cross-linking of collagen fibrils by lysyl oxidases (LOX) to form an insoluble scaffold, which helps support tissue structure [23,24]. The network-forming collagen IV is highly present in basement membranes. Adhesive glycoproteins such as fibronectin and tenascins are detected in the subcapsular connective tissue, septa, and portal areas, and fibronectin is the main ECM component in Disse’s space in normal liver [25]. Proteoglycans (PGs, e.g., lumican and fibromodulin) act as “space fillers” of the ECM and function in the assembly of collagen fibrils; they are formed by a core protein onto which several glycosaminoglycan (GAG) chains are covalently attached (heparin, heparan, dermatan, keratan, and chondroitin sulfate) [26]. Low amounts of elastin are also present in this interstitial matrix, increasing in diseased liver [27]. The basement membrane of the liver ensures a scaffold for the attachment of hepatocytes and endothelial cells, and its loose structure allows for the rapid diffusion of small molecules. It is a prominent reservoir of angiogenic growth factors and enzymes that control biological processes such as ordered cell migration and adhesion, wound healing, and tissue regeneration. It is composed of the network-forming type IV collagen, laminins, specific PGs containing mainly heparan sulfate (HSPG), and nonscaffolding collagens such as the perisinusoidal collagen type XVIII mainly produced by hepatocytes [28] or collagen type XV located in the portal tract. Type IV collagen is produced by endothelial cells and forms a 3D network instead of fibrils, ideally suited for the incorporation of laminins and proteoglycans. This network forms a low-density matrix along the sinusoids, bile ducts, and vessels of the portal tract. This helps maintain the differentiated and polarized functions of the cells attached to it, notably hepatocytes and cholangiocytes. Type IV collagen can be degraded by matrix metalloproteases to give rise to subdomains with signaling capability, known as matrikines or matricryptins such as tumstatin [29]. Other components of the hepatic ECM comprise GAGs and hyaluronic acid or hyaluronan (HA), a nonsulfated GAG not attached to a core protein.

**Figure 1:**
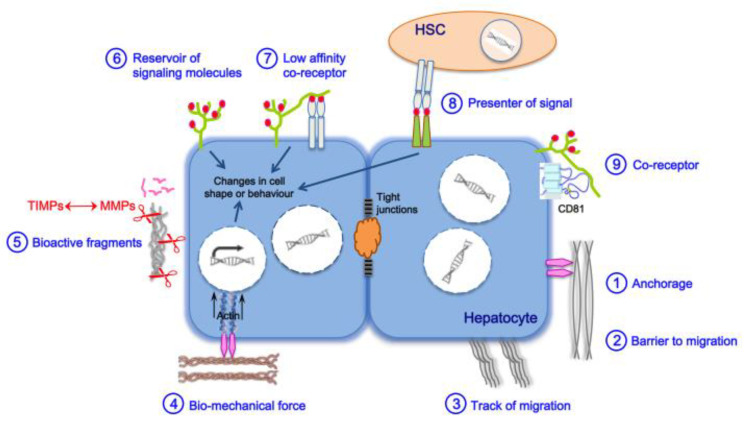
Mechanisms of ECM functions. Biological functions of the ECM are related to its biochemical and biomechanical properties. ① Anchorage to the basement membrane is essential for various processes, such as maintenance of polarity, cell proliferation, and differentiation. ②③ The ECM may also serve to block or guide cell migration. ④ Cells are able to sense the biomechanical properties of the ECM (e.g., stiffness), and change their shape or behavior through mechanotransduction pathways: tensional forces, focused within focal adhesion structures, induce clustering of integrin receptors, which causes recruitment of signaling proteins such as talin, vimentin, paxillin, tensin in direct connection with actin cytoskeletal filaments and microtubules. Several kinases also concentrated at the focal adhesion transfer stimuli from the ECM to intracellular signaling cascades; all these events will ultimately contribute to genome transcription and protein translation. ⑤ The ECM directs signals to the cell through bioactive fragments after their processing by proteases such as MMPs, regulated by TIMPs. ⑥ The ECM acts as a reservoir of signaling molecules by binding and by locally concentrating growth factors, cytokines, and hormones. Some ECM components such as HSPGs can selectively bind to different growth factors and function as low-affinity coreceptors ⑦ or as presenters of signals between hepatocytes and HSCs ⑧, thereby playing a major role in cell–cell communication. ⑨ We demonstrated that the HSPG syndecan-1 and the tetraspanin CD81 interact together; this interaction tightly links the ECM, the tetraspanin web, and likely the cytoskeleton and could have functional consequences on both cell behavior and ECM remodeling. Syndecan-1/CD81 form a coreceptor complex for HCV entry [31].

ECM functions are schematically represented in Figure 1 and can be roughly divided into physical and biochemical properties. In terms of physical properties, the hepatic ECM plays a role in the anchorage of liver cells to confer cohesion to the epithelium (to establish and maintain cell polarity). Hepatic ECM is also a physical barrier to cell migration or conversely can direct this migration through the organization of collagen fibrils in bundles. Liver cells are capable of reacting to biomechanical properties of the hepatic ECM through mechanosensing/-transduction machinery involving the focal adhesion complex and the actin cytoskeleton and ensuring a continuum for signals to propagate from the ECM to the nuclear chromatin. In particular, matrix stiffness and fibrillar architecture can be sensed, generating signals translated into changes in cell shape or behavior [30].

Biochemical properties of the hepatic ECM include: (i) its ability to capture and bind to growth factors, cytokines, and chemokines and locally concentrate them at the cell surface, thereby acting as a reservoir of signaling molecules. ECM proteoglycans such as heparan sulfate proteoglycans (HSPGs) of the hepatocyte membrane can also bind molecules and function as low-affinity coreceptors or as signal presenters for another cell type present in the space of Disse, with an important role in intercellular communication; (ii) its capacity to send signals to cells, triggered by bioactive fragments of its protein components such as matrikines and matricryptins [29,32], after their processing by MMPs; these processes are regulated by a finely tuned balance between MMPs and TIMPs.

A tight intricacy also exists between the hepatic ECM and cells residing within, with reciprocal interactions contributing to liver homeostasis. Therefore, if any of the physical and biochemical properties of the hepatic ECM are altered, abnormal behavior of cells of the connective tissue will occur, leading with time to the disruption of liver homeostasis and to functional failure observed in fibrosis and cirrhosis.

## 3. Liver Fibrosis and Cirrhosis

### 3.1. General Pan-Etiology Features

**Figure 2:**
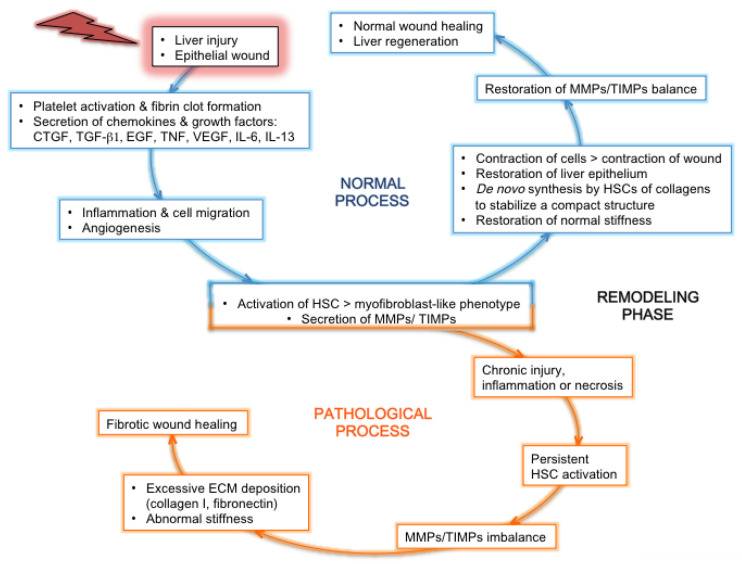
Normal or pathological process after liver injury: tissue regeneration or fibrosis. After the initial event of liver injury, the epithelial wound accompanied by a breach in the endothelium triggers the coagulation cascade, followed by an inflammatory and proliferation phase mediated by the secretion of inflammatory cytokines and growth factors. The profibrotic cytokines IL-13 and TGF-β1 are secreted by activated leukocytes coming from the blood circulation and by sinusoidal cells [36]. Concomitantly, HSCs are activated, thereby adopting a myofibroblast-like phenotype and secreting MMPs and TIMPs. These proteins contribute to ECM remodeling, together with cytokines and chemokines that recruit leukocytes at the site of injury and activate them. In the loop of a normal wound-healing process (**blue**), the inflammatory process gives way to a progressive tissue repair, with the cleaning up of tissue debris and dead cells by leukocytes, the contraction of epithelial cells to restore a normal epithelium, and the de novo synthesis by HSCs of ECM components that organize in order to stabilize a compact structure between and around cells. This helps to restore normal stiffness. In parallel, endothelial cells form new blood vessels. The balance of secretion and activity between MMPs and TIMPs is restored to normal. All these features lead to normal wound healing and liver regeneration. In the loop of a pathological/fibrotic wound-healing process (**orange**), a state of chronic injury and inflammation is maintained, accompanied by tissue necrosis instead of repair. This leads to the persistent activation of HSCs. Thereby, the tight balance between MMPs and TIMPs secretion and activity is disrupted, and overly abundant amounts of ECM components produced by activated HSCs are deposited in the interstitial tissue, which becomes scar tissue with abnormal stiffness. Within this stiffer tissue, the migration of cells and chemokines that could contribute to healing is greatly impaired. Altogether, these features contribute to a fibrotic wound-healing phenotype, with the formation of a permanent fibrotic scar.

The main causes of fibrosis are infections with HCV or HBV, alcohol abuse, and nonalcoholic fatty liver disease (NAFLD). Fibrosis is a reversible exuberant wound-healing and scarring process in which excessive connective tissue builds up in the organ (reviewed in [17] for the liver). This dynamic phenomenon is triggered by a chronic liver injury (Figure 2), which causes an imbalance between excessive ECM production (fibrogenesis) and deficient degradation (fibrolysis), during which several cell types are recruited onsite to help “seal off” the injury [33]. Mature scar ECM, composed of cross-linked collagens and elastin, is more resistant to MMPs, and fibrils sequestered in deeper portions of scar become inaccessible to these enzymes [34]. Whatever the etiology, this injury is linked to impaired hepatocyte replicative capabilities, including hepatocyte death, and activates HPCs, i.e., triggers their proliferation and differentiation [35]. This process helps provide new hepatocytes and maintain the organ’s functional integrity and is accompanied by liver inflammation. Cirrhosis is characterized by the disruption of the normal hepatic architecture, with a distortion of the blood flow through the liver: tissue septa form, which connect the incoming vasculature (portal vein and hepatic artery branches) and outgoing vessels (central veins). This may lead to portal hypertension and is accompanied by inflammation, angiogenesis, and hepatic endothelial dysfunction, leading to a global liver dysfunction.

Staging of fibrosis is based on liver biopsy and/or noninvasive methods measuring liver stiffness (transient elastography). One of the most commonly used tools to evaluate the severity of chronic liver disease is the METAVIR score, delineating four stages: F0, normal liver; F1, portal fibrosis without septa; F2, portal fibrosis with few septa; F3, numerous septa without cirrhosis. Liver cirrhosis, the most advanced stage, is defined as F4 [37]. Serum biomarkers may be further analyzed as indicators of a higher risk of fibrosis. These markers can be classified as indirect (a combination of routine liver biochemistry and general features) or direct (a reflection of liver extracellular matrix turnover and accumulation). Direct markers include soluble components of the ECM (HA, tissue inhibitor of matrix metalloproteinase-1, collagen byproducts; see Section 3.2.1 and Section 3.2.2). Liver inflammation related to fibrosis also involves liver-resident macrophages and peripheral monocytes (see Section 2). From that, the frequency of CD14+ monocytes was found significantly higher in HCV fibrotic patients than in healthy individuals and positively correlated with liver fibrosis. Serum levels of CD163, a marker of liver macrophage subpopulation, also correlated with HCV-related liver fibrosis and was proposed as a novel marker for assessing the degree of liver fibrosis in HCV-infected patients [38].

As initial stages of fibrosis are asymptomatic, the diagnosis could be delayed, with delayed implementation of therapy. Indeed, a successful resolution of fibrosis largely depends upon the stage and extent of scarred tissue. Treatments involve correcting the underlying condition when possible, e.g., eliminating excess alcohol consumption, changing to a healthier lifestyle in NAFLD patients, and administering appropriate antiviral therapies to patients with viral hepatitis. This is also valid in the cirrhotic range of fibrosis; de facto, as long as liver functions are maintained, cirrhosis is no longer termed as end-stage disease but as advanced liver disease. However, its therapeutic resolution is more difficult to obtain than that of earlier stages of fibrosis [37], and cirrhotic patients run a 1–7% yearly risk to develop HCC [33].

### 3.2. Features Linked to HCV Pathogenesis

HCV leaves the circulation through the fenestrae of the sinusoid capillaries and crosses the space of Disse. HCV infection of hepatocytes occurs after recognition at the cell plasma membrane of a quartet of receptors necessary and sufficient for viral entry: the tetraspanin CD81, the scavenger receptor SR-BI, and the components of tight junctions claudin-1 and occludin [39]. Recently, we identified the heparan sulfate proteoglycan (HSPG) syndecan-1 as a cofactor of CD81 for HCV entry [31]; both molecules form a complex linking the ECM to the cytoskeleton [40] and integrins, receptors of ECM components [41]. This emphasizes the subtle connection that occurs early between HCV infection, hepatic ECM, and key components of the intracellular machinery that could act as sensors of ECM physical properties (stiffness/tension; Figure 1).

Persistent HCV infection of hepatocytes induces the activation of the focal adhesion kinase, leading to increased expression of paxillin and delocalization of α-actinin [42], forming the focal adhesion complex [43]. This might translate into modifications of cell adhesion and migration properties and trigger cytoskeletal reorganizations transduced into signals transiting to the nucleus through mechanotransduction machinery (Figure 1). HCV-mediated liver fibrogenesis appears at portal and hepatocellular sites, with ECM deposition around sinusoids in the vicinity of the portal vein as well. This is in contrast with perivenular and perihepatocellular fibrosis, with ECM deposition in the space of Disse, observed in NAFLD- or alcohol-related fibrosis [44,45]. Other specific clinical signs of HCV-related fibrosis include the clustering of mononuclear cells at the hepatic lobules and the presence of prominent aggregates of lymphocytes in periportal zones [46,47] as indications of a major inflammatory activity not observed in alcohol-related fibrosis [46], also reported in the recently developed rat model of the hepatitis C-like virus [48]. Such clusters of liver-resident macrophages play a key role in liver inflammation through the secretion of proinflammatory cytokines and chemokines [49]. Additional discriminating features include prominent steatosis in HCV-infected hepatocytes [48], as a result of HCV-mediated metabolic reprogramming and necroinflammation, more commonly observed in chronic hepatitis C than B [50] but less than in alcohol-related liver disease [44]. Bile duct damage is also more observed in chronic hepatitis C than B [51]. Bile ductular reactions originate from cholangiocytes or hepatocytes and accompany cholestatic liver diseases such as cholangitis, as well as parenchymal liver cell diseases induced by alcohol and HCV or HBV infections. These reactions are often linked to fibrosis and portal inflammation in chronic liver diseases. During fibrosis, bipotent HPCs produce an excess of fibrogenic mediators, such as transforming growth factors (TGFs) TGF-β1 and -β2, platelet-derived growth factor (PDGF), connective tissue growth factor (CTGF), and sonic hedgehog, supporting HSC proliferation and activation [33]. Interestingly, transcriptomic analyses of HPCs from patients with advanced fibrosis/cirrhosis linked to cholangitis or chronic hepatitis C revealed patterns of gene expression differing in disease etiology [47]. Progenitors from cholangitis patients showed enrichment in morphogenesis and cytoskeleton organization markers, whereas cells from hepatitis C patients displayed an increase in metabolism/hepatocyte markers and networks enriched for cell movement and receptor activity. Ductular reactions in HCV-mediated liver disease were also associated with intense vascular remodeling not observed in cholangitis. Chronic hepatitis C causes major changes in the inflammatory cytokine and chemokine milieu, susceptible to be translated into specific disease manifestations [52]. This agrees with the fact that HCV-associated progenitors and their niche display an increase in invasion- and metastasis-related markers, such as PDGF-α [53] and the insulin-like growth factor-2 [54]. This reveals a striking similarity with cancer progression, i.e., invasion into the parenchyma and (neo)angiogenesis. Additionally, PDGF-α is a profibrotic actor, as it activates HSCs, thereby contributing to the biosynthesis, secretion, and deposition of components of the ECM [55].

The hepatocyte nuclear factor HNF4α is a transcriptional regulator of glycogen metabolism, cell junctions, differentiation, and proliferation in liver and intestinal epithelial cells; it is essential for hepatocyte differentiation during embryogenesis. HPCs from cirrhotic HCV-patient biopsies exhibited nuclear foci of HNF4α, whereas the transcriptional factor c-Jun was more expressed in cells from cholangitis patients [47]. This indicates an etiology-dependent activation of specific transcriptional regulators, HPCs being primed or pushed toward a certain cell fate. In the case of HCV-mediated chronic liver disease, HPCs are therefore pushed toward hepatocytes instead of cholangiocytes [47]. On the path to hepatocellular carcinoma, HPCs are on the contrary maintained in their undifferentiated state, and pushed toward stemness (self-renewal and expansion), under the influence of the lectin galectin-3, as well as α-ketoglutarate, a compound derived from glutamate, both secreted by transformed hepatocytes [56]. Galectin-3, like PDGF, is an activator of HSCs; both molecules therefore play a dual role in HCV pathogenesis at early (fibrosis/cirrhosis) and later (oncogenic transformation) stages of liver disease.

Concerning the ECM, which features could be attributed to HCV-mediated liver disease, possibly linked to the expression of viral proteins? During this pathology, a profibrotic phenotype is acquired, with increased expression and release in the connective tissue of collagens I and IV [55,57,58], elastin [59], proteoglycans such as fibromodulin [60] or lumican [59], and HA [42]. These overly expressed components, together with dysfunctions of enzymes involved in their metabolism, contribute to alterations in the properties of the hepatic ECM during chronic infection. A spectrum of expression of these and other ECM constituents, enzymes, and regulators of the ECM will therefore be analyzed in the following, in connection with the expression of HCV proteins when identified. Reported connections between HCV proteins and elements of the ECM or cytokines are summarized in Table 2. Correlations between ECM proteins or cytokines expression and METAVIR liver disease/HCC stages in chronically HCV-infected patients are reported in Table 3.

#### 3.2.1. Collagens and Derived Fragments

By specifically analyzing the ECM components of biopsies from HCV-infected patients with liver fibrosis, levels of collagens I, III, and V were found increased from stages F0/F1 to F4 [59,79,80]. However, this is a hallmark of all fibrotic diseases [102]. These fibrillar collagens form arrays incorporating fibril-associated collagens with interrupted triple helices (FACIT), such as collagens XII and XIV [114]. Hepatic collagen XII amounts decreased with increasing stages of HCV-related fibrosis, whereas collagen XIV levels increased [59,84]. Collagen XVI, another FACIT not associated with collagens I, III, or V fibrils [115], was found underexpressed, especially at the cirrhotic stage of HCV-related fibrosis [59]. Collagen XVII is a transmembrane protein and a main component of hemidesmosomes, which are cell–ECM junctions anchoring epithelial cells to the basement membrane by interacting with integrins outside and with intermediate filaments of the cytoskeleton inside the cells [116]. Patients with advanced fibrosis/cirrhosis linked to chronic hepatitis C revealed focal positivity for collagen XVII in sinusoidal lining cells and few cholangiocytes, a pattern not observed in patients with cholangitis-linked cirrhosis [47]. Collagen XVII was also observed in the cytoplasm of HPC, and in the surrounding basal membrane in end-stage HCV-linked fibrosis. Collagen XVIII is a basement membrane collagen almost exclusively expressed in the liver, and hepatocytes were identified as its main source [28,117]. It can be cleaved at its C-terminus to generate endostatin, a powerful antiangiogenic agent [117,118]. Collagen XVIII was found underexpressed in HCV-diseased liver [59], and through the subsequent downexpression of endostatin, hepatocytes could play an unforeseen role in neoangiogenesis during hepatic neoplasia.

Studies attempting to define etiology-dependent molecular signatures of liver fibrosis identified serum collagen-derived biomarkers that variably evolve during chronic hepatitis B and C. Pro-C3 (N-terminal type III collagen propeptide), a fragment of collagen III formation, has been proposed as a serum predictive biomarker of fibrosis progression in patients with chronic hepatitis C [85]. These patients displayed higher serum levels of this biomarker than HBV-chronically infected patients [50]. By contrast, serum of HBV-infected patients had higher levels of the protease-cleaved 7S domain of the amino-terminal propeptide of type IV procollagen P4NP7S, a biomarker of type IV collagen formation. Chronic HBV-infected patients also had higher serum levels of markers of collagens III, IV, and VI degraded by matrix metalloproteases than chronic HCV patients as a reflection of a greater basement remodeling induced by chronic hepatitis B [50].

#### 3.2.2. Enzymes of the ECM

##### 3.2.2.1. Lysyl Oxidases

The lysyl oxidase family of enzymes comprises five members: LOX and LOX-like 1 to 4 (LOXL). They are copper-dependent secreted amine oxidases that cross-link monomers of collagen or elastin to form insoluble fibrils. Hepatocytes of healthy livers do not express or express only low amounts of lysyl oxidases [119]. In viral hepatitis C, LOX and LOXL1 expression is strongly enhanced in activated HSCs but not in hepatocytes [119]. Likewise, in viral hepatitis, collagen deposits are only observed in fibrotic zones and not around hepatocytes, in contrast to what is observed in fibrotic liver diseases of other etiologies. LOXL2 is strongly induced in fibrotic liver, where it localizes to regions of the collagenous matrix and α-SMA-positive fibroblast-like cells [120]. More specifically, in fibrotic liver diseases related to active hepatitis C infection, LOX and LOXL2 were detected at the fibrotic disease interface composed of fibroblasts, hepatocytes, and neovasculature [120]. LOX was found to contribute to collagen stabilization in liver fibrosis, promote fibrogenic activation of HSCs, and limit fibrosis reversal [121]. LOXL2 also mediates fibrotic matrix stabilization and stimulates differentiation of HPCs toward fibrogenic cholangiocytes [122]. The fibrosis-promoting activities of LOX and LOXL2 are susceptible to occur even after cessation of the chronic liver injury. Together with the global morphological reorganization of the liver due to ECM accumulation that alters liver metabolism, these impaired enzymatic activities might contribute to the persistence of clinical signs of liver disease after SVR in the case of chronic hepatitis C, in the absence of any therapy targeting the enzymes [120,123]. This feature may be even more pronounced when alcohol intake and/or metabolic syndrome complicate chronic infection. Thrombospondin-1 belongs to the family of matricellular proteins, which mediate cell–cell and cell–ECM interactions but are not primary structural ECM elements. It plays a role in collagen homeostasis, through its binding to fibrillar collagens, pro-LOX, matrix metalloproteases (MMPs), and TGF-β1 [71,72]. The core protein of HCV was found to downregulate LOX while upregulating the genes of collagen I (*COL1A1*) and thrombospondin-1 (*THBS1*) [73]. Similar gene expression profiles were obtained from mice xenograft tumors derived from HCV-infected human hepatocytes [74]. Mechanistically, HCV, and core in particular, activate the production of latent TGF-β1 by infected hepatocytes [53,73,74]. After its secretion in the microenvironment, latent TGF-β1 is then cleaved into active TGF-β1 by thrombospondin-1, the expression of which is upregulated by HCV [75], and the core protein in particular [73]. Active TGF-β1 finally activates hepatic stellate cells, which contribute to fibrogenesis by (over)producing ECM components [73]. Interestingly, *THBS1* is a TGF-β1 target gene, which creates a vicious loop where activated HSCs are committed to ECM overproduction.

##### 3.2.2.2. Matrix Metalloproteases (MMPs) and Their Inhibitors

Under physiological conditions of liver regeneration during wound healing, the normal equilibrium between MMPs and their inhibitors (tissue inhibitors of MMPs, TIMPs) is restored, whereas the MMP/TIMP ratio remains imbalanced during fibrosis (Figure 2). The mRNA and protein expression of several MMPs is modified during chronic hepatitis C: the gelatinases MMP-2 and -9 are enhanced [57,58,76], while the collagenases MMP-1 and -13 are downregulated [57]. A recent study reported increased serum levels of MMP-2, -7, and -9 in patients with chronic hepatitis C, which correlated to the fibrosis stage for MMP-7 [90]. Gelatinases contribute to the degradation of the basement membrane collagen IV, while collagenases such as MMP-1 and -13 break down interstitial collagens I and III. During chronic hepatitis C, the upregulation of gelatinases will lead to a major reshuffling of the basement membrane, with the destruction of the structural support of cells, and the downregulation of collagenases will amplify the deposition of insoluble collagen fibers and aggravate the fibrotic phenotype. As mentioned earlier, hepatic collagen XIV is increased during HCV-related fibrosis [59,84]; as this collagen is collagenase-sensitive, this observation is in line with the downregulation of collagenases [57].

In parallel, the expression and secretion of TIMPs, in particular TIMP-1, are enhanced to compensate for the increased activity of MMPs [57,124]. Elevated levels of MMP-2 and -9 were reported in the serum and/or liver specimens of chronic hepatitis C patients, correlating with the fibrosis stage but not with the viral load [63,76,88]. The serum level of TIMPs was suggested as an indicator of hepatic fibrosis: increased serum levels of TIMP-1 in chronic hepatitis C patients correlated with the stage of liver fibrosis [86], better than those of TIMP-2 [91]. Interestingly, serum levels of MMP-9 and TIMP-1 decreased after SVR in fibrotic HCV patients treated with DAAs, in parallel with the regression of fibrosis [125]. When fibrosis etiologies are compared, it appears that MMP-10 and -11 are upregulated at late fibrosis stages of chronic hepatitis B, whereas at similar stages of chronic hepatitis C, MMPs-2 and -9 are upregulated [89]. In NAFLD and nonalcoholic steatohepatitis, MMP-9 and -10 were found at significantly higher levels than in chronic viral hepatitis B and C [89].

To study the underlying mechanisms of such enzyme involvement in HCV-related fibrogenesis, hepatoma cell lines stably expressing the HCV protein(s) of interest were generated. The highest levels of MMP-2 were secreted when primary HSCs were grown with hepatoma cells expressing HCV core, compared with HSCs grown alone or in the presence of “regular” hepatoma cells [58]. Hepatocytes expressing HCV core secrete high amounts of TGF-β1 and exhibit a transcriptional upregulation of the connective tissue growth factor (CTGF), a mitogen stimulating the production of ECM components and enzymes by HSCs. This suggests that HCV core is a key regulator of MMP(-2) expression, possibly through a TGF-β1-mediated upregulation of CTGF [58]. Along similar lines, Chang liver cells expressing HCV core displayed elevated levels of MMP-9 transcript and protein not observed in NS5A-expressing hepatocytes [63]. This suggests a direct effect of the core protein on the regulation of MMP-9 protein synthesis and could explain the elevated levels of hepatic MMP-9 in biopsies from patients with HCV not observed in patients with nonalcoholic steatohepatitis. Interestingly, both core- and NS5A-expressing cells displayed elevated levels of cyclooxygenase-2 (COX-2), a key enzyme of the biosynthesis of prostaglandins, mediators of inflammation. High hepatic levels of COX-2 were also reported in HCV-patient biopsies, which places core and NS5A at a regulatory hub between inflammation and fibrogenesis [63]. This is reminiscent of a strong correlation between the inflammatory activity of the liver of fibrotic HCV patients and the transcriptional levels of TIMP-1 [88]. Similar higher levels of MMP-9 were reported in the serum of chronically HCV-infected patients and in HCV-infected hepatoma cells than in uninfected patients or cells, and MMP-9 displayed greater enzymatic activity [71]. COX-2 was also found overexpressed in HCV-infected hepatocytes. Mechanistic studies revealed a regulatory role of the HCV serine protease NS3, together with its cofactor NS4A, underlying such overexpression and activity: HCV NS3 activated the ERK1/2–p38 pathway, leading to the phosphorylation of ERK1/2 and p38. This led to nuclear factor-κB (NF-κB) activation, and translocation to the nucleus where it promoted *MMP9* transcription, leading to MMP-9 (over)expression [83]. In parallel, NF-κB promoted *COX2* transcription, and once biosynthesized, COX-2 translocated to the nucleus, where it acted as a transcriptional promoter of *MMP9*. COX-2 and MMP-9 contributed to inflammation and fibrosis through the synthesis of prostaglandins and ECM degradation, respectively [71]. As noticed earlier, HCV-mediated fibrosis appears tightly linked to liver inflammation, with on the one hand viral proteins (core, NS3, NS5A) acting as modulators of inflammation pathways and on the other hand inflammation mediators such as COX-2 acting as regulators of liver fibrosis. This is also in line with the anatomical observations of the gathering of mononuclear cells at the hepatic lobules in HCV-related fibrosis, a hallmark of inflammatory activity not observed in alcohol-related fibrosis [46], and the presence of prominent aggregates of lymphocytes in periportal zones [46,47]. The nonstructural protein NS4B was also identified as an activator of MMP-2 expression, both at the mRNA and protein levels [76]. Mechanistic studies revealed that NS4B activated the ERK/JNK pathway, which engaged the transcription factor STAT3, thus contributing to *MMP2* transcription.

##### 3.2.2.3. A Disintegrin and Metalloprotease with Thrombospondin Motifs (ADAM and ADAM-TS)

ADAMs, comprising ADAM-TS, are membrane-bound or secreted zinc proteases of the ECM, with a broad tissue distribution. Through their disintegrin and metalloprotease domains, they are therefore able to carry out cell adhesion and protease activities, respectively. In the liver, they are involved in the regulation of epithelial cell regeneration after liver injury and able to directly degrade ECM components, thereby promoting ECM rearrangement during wound healing and fibrosis. A strong correlation between ADAM-9, -28, and -TS1 vs. MMP-2 and α-SMA was identified in biopsies from patients with diseased liver of any etiologies [126]. No differences in ADAM expression were detected in biopsies of different etiologies. The expression of ADAM-10 and -17 correlated with the severity of fibrosis in patients with chronic liver diseases [127]. Higher expression of ADAM-TS2 was detected in cirrhotic than in normal liver and correlated with TGF-β1 expression [94], independently of the etiology of liver disease. However, ADAM-TS2 expression is indirectly linked to HCV-induced fibrogenesis. In an attempt to identify noninvasive markers of liver fibrosis during chronic hepatitis C, the Enhanced Liver Fibrosis (ELF) test was put forward; it evaluates the serum levels of TIMP1, HA, and N-terminal peptide of procollagen type III (PIIINP) and includes age for statistical robustness [128]. This test has been shown to properly identify moderate and severe fibrosis in the context of chronic hepatitis C [128], and in particular, the serum levels of PIIINP could differentiate between mild and moderate/high HCV-related liver fibrosis [85]. Because ADAM-TS2 excises the N-propeptide of the fibrillar procollagen types I, II, III, and V, one can infer that an implicit evaluation of ADAM-TS2 expression and activity is contained in the ELF test, in relation to HCV pathogenesis and fibrogenesis.

Recent genome-wide association studies, aimed at identifying genetic polymorphism behind the risk of developing HCV-related HCC, also unveiled a mechanistic scheme linking ADAMs to HCV pathogenesis. A single-nucleotide polymorphism (SNP) was identified in the 5′-flanking region of major histocompatibility complex class I-related chain A or MHC class I polypeptide-related sequence A (*MICA*) as a susceptibility gene for HCV-induced HCC. In these patients, this SNP was associated with progression from chronic hepatitis C to cirrhosis and HCC [129]. MICA is a cell surface glycoprotein of epithelial and endothelial cells, monocytes, and fibroblasts, involved in particular in antiviral defense responses [130]. It functions as an indicator of cellular stress and activates circulating cytotoxic natural killer (NK) cells, key actors of immune surveillance, deploying in particular anticancer activity. MICA expression is induced in cells undergoing oncogenic or viral stress [131]. In the context of HCV infection, membrane-bound MICA (mMICA) expression in HCV-infected hepatocytes was downregulated by the NS3/4A serine protease [74]. Hepatocytes expressing the cysteine protease NS2 or the polymerase NS5B exhibited decreased MICA expression when cocultured with NK cells [70], supporting the notion that HCV NS2 and NS5B disable MICA in infected hepatocytes, thereby inhibiting the ability of these cells to respond to stimuli from NK cells. MICA is shed from the membrane by ADAM-9, -10, and -17, which generates soluble MICA (sMICA) [132,133,134]. ADAM-mediated shedding of MICA therefore leads to higher levels of sMICA and lower levels of mMICA. Because ADAM-9, -10, and -17 expression increases with the severity of fibrosis in patients with chronic liver diseases [127,131], more MICA shedding occurs in these pathologies, translated into higher levels of sMICA. Interestingly, sMICA is an inhibitor of the activity of NK cells, in particular their activity against HCC [135]. Elevated levels of sMICA were observed in patients with advanced HCC, associated with impaired activation of NK cells [131]. In the peculiar context of HCV-induced HCC, patients bearing the allele at higher risk of HCC occurrence displayed higher sMICA levels [129]. This correlated with a poorer prognosis, and a higher risk of developing liver disease in a context of escape from NK-mediated immune surveillance [129,131]. Finally, a correlation was found between high sMICA levels, the higher-risk allele, and the development of HCC in HCV-infected cirrhotic patients who failed to develop a SVR [136]. Of note, therapies combining DAAs and inhibitors of ADAMs are currently debated for this type of patient [131,135].

#### 3.2.3. Proteoglycans

Proteoglycans (PGs) are membrane-associated and soluble proteins of the ECM. They are composed of long chains of sugar molecules, anchored on a short polypeptide chain. Sugars can represent up to 90% of their weight. This is in contrast to matrix glycoproteins, comprising small sugar chains anchored to a long polypeptide chain and where sugars do not represent more than 60% of their weight (see Section 3.2.5). Through their GAG chains, PGs interact with numerous regulatory molecules, such as cytokines, growth factors, and hormones. This has repercussions on a myriad of normal and pathological cellular processes. PGs are classified as syndecan-like integral membrane PGs (SLIPs), glypican-related integral membrane heparan sulfate PGs (GRIPs), membrane-associated β-glycan and CD44, extracellular small leucine-rich PGs (SLRPs), and hyalectans [137].

##### 3.2.3.1. Membrane-Associated PGs

The SLIP family of PGs comprises four molecules, syndecans-1 to 4. Syndecan-1 harbors heparan- and chondroitin-sulfate GAGs (HS and CS, respectively), while other syndecans only bear HS. Interestingly, syndecan-1 and -4 were identified as cofactors of HCV attachment and entry into hepatocytes [31,138,139]. Mechanistically, syndecan-1 forms with the tetraspanin CD81, a complex internalized with virions during viral entry. HCV core and syndecan-1 colocalized during the intracellular trafficking of virions [31]. When chronic infection of hepatocytes was established, syndecan-1 expression was downregulated [31]. This is in line with observations of biopsies collected from patients with HCC, including HCV-infected patients [140]: the downregulation was more pronounced in the tumoral tissue than in peripheral nontumoral zones and correlated with the aggressiveness of the tumor. However, these data remain controversial, as other studies reported an upregulation of syndecan-1 during cirrhosis and in association with HCV-related HCC [141]. Syndecan-1 downregulation correlated with the upregulation of xylosyltransferase-2 [31], a key enzyme located in the ER/Golgi compartments involved in the biosynthesis of the tetrasaccharide linkage region of CS and HS PGs [142]. Xylosyltransferase-2 activity was found increased in the serum of patients with HCV-related liver fibrosis at early stages [95,96], evocative of a dramatic remodeling of PGs within the fibrotic ECM. Of note, this correlation became negative in the cirrhotic range of liver fibrosis [95]. Thus, higher enzymatic activity at the fibrotic stage could be seen as a compensatory mechanism against HCV-mediated liver injury; at the cirrhotic stage, as more damage has occurred, this compensation might become inefficient or disappear.

The GRIP family of PGs comprises the glypicans, anchored to the membrane through a glycosylphosphatidylinositol tail [143]. Among the six glypicans, the most studied is glypican-3 (GPC3), an oncofetal protein widely expressed during development but silenced in adult tissues. It is seen as a negative regulator of cell growth [144], and it is overexpressed in cancers such as embryonic tumors [145] and HCC [97]. In a cohort of HCV-infected patients with liver disease, high levels of GPC3 transcript and protein appeared as good markers of early stages of HCC, discriminating from dysplastic nodules [98]. Moreover, GPC3 was also strongly upregulated in early and advanced HCC compared with normal tissue. As described for syndecan-1, GPC3 forms a complex with CD81. Mechanistic studies revealed the following loops: in resting liver, GPC3 is associated with CD81 at the plasma membrane, thereby preventing CD81 to bind the cytosolic transcriptional repressor hematopoietically expressed Homeobox (Hhex); this repressor therefore migrates to the nucleus, where it exerts its growth inhibitory role. In regenerating liver (notably after an injury), GPC3 binding to CD81 decreases, which leads to increased binding of CD81 with Hhex in the cytosol; less Hhex therefore migrates to the nucleus, and proliferation is boosted [146]. During HCV-mediated liver disease and HCC, the virus, and in particular its membrane glycoprotein E2, which binds CD81, was shown to mimic GPC3 in infected hepatocytes, thereby interfering with the GPC3/CD81 binding [69]. A decrease in GPC3/CD81 entities leads to more CD81/Hhex cytosolic binding, so less nuclear Hhex. This fuels neoplastic proliferation. HCV is thus likely to enhance liver neoplasia by acting as a growth promoter of neoplastic hepatocytes, through its binding to CD81 to the detriment of GPC3 [69].

A remarkable feature of SLIPs and GRIPs is the ability of their ectodomain to be cleaved by ECM proteases in a process called shedding. The resulting soluble domain then acts as an autocrine and/or paracrine signal in the microenvironment per se and via the factors attached to its GAG chains. Elevated serum levels of shed syndecan-1 have been proposed as biomarkers of prediction of liver fibrosis but not of liver inflammation in chronic hepatitis C patients [147]. However, of note, these data should be cautiously taken, as high serum levels of syndecan-1 are also observed during, e.g., inflammation, sepsis, and vascular dysfunctions. Concerning GPC3, plasma levels of the shed form were found higher in patients with HCC than in healthy individuals; they were also higher in patients with HCC induced by HCV than by HBV or other etiologies [148]. The full-length form of GPC3 also assayed in blood samples correlated with tumoral GPC3 expression only in patients with HCV-induced liver carcinoma. GPC3 has now demonstrated its added value as a biomarker of HCC and has become a target of cancer immunotherapy [149]. Indeed, T cells expressing GPC3-specific chimeric antigen receptor (CAR-T) were generated to target and destroy GPC3-positive HCC. Several clinical trials are currently ongoing to evaluate the safety and tolerability of such an immune strategy (e.g., ClinicalTrials.gov Identifiers NCT02395250 and NCT02905188). Elevated serum levels of these shed PGs are an indication of increased shedding, and therefore of increased systemic exposure to the factors retained in the GAG moieties, such as TGF-βs. These cytokines could then exert their fibrogenic and oncogenic effect at a distance from their site of biosynthesis. GPC3 was found to suppress the expression and signaling of TGF-β2, which led to activation of cell growth and cell cycle progression [150]; strategies aimed to suppress GPC3 expression were thus proposed as valuable options for the clinical management of GPC3-positive HCC patients. Concerning syndecan-1, paradoxical data were reported: indeed, retention of TGF-β1 and thrombospondin-1 in its GAG chains was shown to inhibit the conversion of latent TGF-β1 into its active form, thereby decreasing the availability of active TGF-β1 [151]. Due to increased shedding, shed syndecan-1 at higher amounts would enter the circulation together with its latent TGF-β1 cargo. Less activation and availability of TGF-β1 would then translate into less activation of HSCs, and therefore less synthesis of fibrogenic molecules (see Section 3.2.2.1). This is somewhat conflictual with the correlation found between high serum levels of shed syndecan-1 and the prediction of liver fibrosis in chronic hepatitis C patients [147]. However, the precise mechanistic link between HCV pathogenesis and increased PG shedding is still missing, although the HCV-induced enhancement of protease expression could play a role (see Section 3.2.2).

The cluster of differentiation molecule 44 (CD44) is a membrane-associated adhesion glycoprotein of the ECM, considered as a “part-time PG,” as covalent attachment of a CS or HS to its protein chain depends on developmental or pathological cues [152]. It is produced as standard (CD44s) and variant (CD44v) isoforms, and it is the receptor of hyaluronan (HA), a soluble nonsulfated GAG (see Section 2). Specific interactions between CD44 and HA are of major importance for the maintenance of the proper (stem cell) niche properties under physiological conditions and the biology of cancer stem cells under pathological circumstances [152]. Together with other cell surface molecules, CD44 was proposed as a marker of cancer stem cells in HCCs, which are responsible for tumor progression and aggressiveness. In particular, CD44 expression in HCCs was related to the TGF-β-mediated regulation of the epithelial-to-mesenchymal transition [153], and its level of expression in tumoral tissue was associated with negative prognosis [154]. In HCV-induced HCC, Variant 9 of CD44 was identified as a biomarker of liver cancer stem cells, and its expression correlated with tumor invasiveness and poor patient survival [155]. Interestingly, CD44v9 expression also correlated with the expression of markers of liver fibrosis (fibronectin) and HSC activation (myosin light chain), suggesting that CD44v9 could play a role at early stages of the HCV-induced liver disease. In patients with chronic hepatitis C, high plasma levels of HA were associated with the progression of liver fibrosis, whereas low serum levels of the interferon gamma-inducible protein 10 (IP-10) correlated with a favorable outcome to anti-HCV therapy [156]. IP-10 is a biomarker of liver inflammatory activity, correlating with lobular fibrosis and necroinflammation but not with portal inflammation or fibrosis. From these observations, Matsuura and coworkers identified a mechanistic loop involving CD44. HCV-infected hepatocytes were found to secrete high amounts of IP-10 in response to the engagement and activation of the innate immune toll-like receptor 2 (TLR2). This secretion depended upon the stimulation of TLR2 by HA and induced an enhancement of CD44 expression. HA was then also able to bind CD44 and further stimulate the production of IP-10. Additionally, CD44 was found to directly interact with TLR2 through its extracellular domain. The mechanistic loop is therefore as follows: high amounts of endogenous HA generated during HCV infection stimulate TLR2 in HCV-infected hepatocytes, which induces the production of the proinflammatory factor IP-10; IP-10 in turn stimulates the expression of CD44, which, as a receptor of HA and a coreceptor of TLR2, will enhance the cellular response to HA stimulation in a vicious loop aggravating the inflammatory profile [156]. Moreover, CD44 acted in synergy with TGF-β1 at early stages of cell transformation to confer stem properties to transformed cells [153].

Another mechanistic loop underlying HCV pathogenesis and involving CD44 was recently described [64]. HCV replication in hepatocytes induced the endogenous expression of osteopontin, a matricellular protein. Osteopontin then localized at the ER, where it contributed to HCV replication, assembly, and infectivity by direct binding to the HCV proteins core, NS3, NS5A, and NS5B. A pool of osteopontin was also processed by proteolysis inside HCV-infected hepatocytes and secreted in the microenvironment, where it exerted autocrine and paracrine signaling through integrin αVβ3 and CD44 binding. This latter binding activated the focal adhesion kinase, which contributed to HCV replication and HCV assembly. Further data indicate that HCV replication was particularly enhanced in liver cancer stem cells expressing CD44 and epithelial cell adhesion molecules (EpCAMs) and that osteopontin also activated HCV replication in these cells [65]. It did so by regulating the stemness of the CD44^+^/EpCAM^+^ cells, thereby inactivating interferon signaling and fueling HCV replication. CD44 is therefore a major actor of HCV pathogenesis, and together with its ligand osteopontin, emerges as a key regulator of cancer stem cells and HCV replication [65]. Recent studies dealing with CD44 also revealed that HCV would replicate at higher rates in liver cancer stem cells than in differentiated hepatocytes, which is of utmost importance for the comprehension of HCV-induced liver disease, notably HCC.

##### 3.2.3.2. Soluble Extracellular PGs

These PGs are classified as extracellular SLRPs and pericellular PGs and hyalectans. SLRPs are extracellular PGs and represent the largest family of PGs, characterized by a small core protein comprised a central region of leucine-rich repeats [143]. Most SLRP members carry chondroitin-, dermatan-, or keratan-sulfate chains, but a few lack any GAG. The main SLRPs comprise biglycan, decorin, asporin, fibromodulin, and lumican. Pericellular PGs such as the heparan sulfate PGs perlecan and agrin are the main components of basement membranes; in the liver, they surround blood vessels and the biliary compartment (mature bile ducts and the HPC niche) in portal zones [143,157,158]. They bind collagens (especially type IV), fibronectin, and laminins (see Section 3.2.5), thereby assisting collagen fibrillogenesis and playing a key role in ECM stabilization and in the maintenance of the functional status of mature connective tissues. Hyalectans (also known as lecticans) are components of the pericellular or interstitial matrix, residing in close vicinity of the cell membrane, important for the communication between cells and ECM, as well as for the regulation of proliferation and migration. They form a ternary binding complex with HA and the matricellular protein tenascin-R, which creates large aggregates in the ECM. The four members of the hyalectan family are versican, aggrecan, brevican, and neurocan [143,159].

The SLRP biglycan is involved in collagen fibril assembly in an ECM-bound form. After proteolytic cleavage, it is released as a soluble form. Proteomic analyses of the ECM of liver biopsies of chronic hepatitis C patients with liver fibrosis revealed a downregulation of the ECM-bound form of biglycan [59]. However, serum levels were found not significantly altered in HCV patients compared to normal controls [160]. Biglycan is a CD44 ligand and also binds the innate immune receptors TLR2 and TLR4 [161]. It is generally considered as a proinflammatory molecule; however, its role and the explanation behind its downregulation during HCV-induced liver disease remain unclear. As a biglycan, decorin plays a role in collagen fibrillogenesis (its name comes from the fact that it “decorates” collagen I fibrils). It binds growth factors, notably TGF-β1. The Glisson capsule of the liver is strongly enriched in decorin. During HCV-induced liver disease, decorin deposition was identified as an early and sensitive indicator of active ECM remodeling after liver injury [92]. This feature correlated with increased TGF-β1 expression for low to moderate stages of fibrosis. However, in this study, a comparable pattern was observed for liver disease induced by other etiologies. As a biglycan, decorin is able to modulate TLR2- and TLR4-mediated signaling and play a role in inflammation; however, our knowledge about decorin and (HCV-induced) liver fibrosis remains piecemeal [162]. Fibromodulin and lumican are SLRPs decorated with keratan-sulfate GAG chains and participate in collagen fibril assembly. Both molecules are biosynthesized by HSCs and are weakly expressed in normal liver [60]. Fibromodulin transcript and protein were found overexpressed in patients with HCV-induced cirrhosis, in parallel with collagen I expression; however, in this study, a similar overexpression was observed for other etiologies of liver fibrosis. Mechanistic analyses revealed that the overproduction of fibromodulin was under the control of oxidative stress, locally induced by the liver injury [60] (and by HCV-induced liver injury in particular [163]). Lumican was also found overexpressed in liver fibrosis [102], and in HCV-induced fibrosis in particular, on liver biopsies [84] and specifically in the ECM [59]. Lumican expression correlated with the severity of the (HCV-related) fibrosis [81,84,102], and high levels of lumican were reported in the plasma of HCV-positive patients with liver disease [81,84]. However, the exact roles of fibromodulin and lumican in (HCV-related) liver disease remain to be determined.

Of the four hyalectans, only versican exhibits a wide distribution; brevican and neurocan are only expressed in neural tissue and aggrecan in cartilage. Versican biosynthesis is stimulated by TGF-β1 and PDGF [143]. In the ECM, it interacts with matricellular proteins, fibronectin, and chemokines via its core domain or GAG chains. It also binds CD44, integrin β1, and at the surface of macrophages toll-like receptors; versican is therefore involved in key cellular functions such as adhesion, proliferation, migration, and invasion. It has been reported as a good biomarker of advanced liver fibrosis in patients with HCV infection or NAFLD, as its hepatic mRNA and serum levels were higher at advanced stages of the disease than at earlier ones [93]. Concomitantly, the levels of ADAM-TS1, involved in versican proteolysis, were enhanced and higher in liver fibrosis than without steatosis [134]. Extracellular vesicles are nanosized particles shed into body fluids by many cell types that carry various bioactive molecules; among them, plasma microvesicles (100–1000 nm in size) are key messengers of cellular communication. Enhanced microvesicle levels were linked to disease activity and progression, e.g., microvesicles isolated from patients with HCV-related decompensated cirrhosis induce vascular hypocontractility, contributing to portal hypertension and circulatory dysfunctions [164]. Versican-positive microvesicles were found elevated in patients with HCV-induced cirrhosis, and further increased with HCC [103]. In patients who had received DAAs, the levels of versican-positive microvesicles dropped at the end of treatment and remained low throughout the 48-week follow-up [164]. This supports the view that DAA-induced eradication of HCV could promote a reversal of fibrosis, and versican-positive microvesicles could be a potential early biomarker of liver fibrosis.

#### 3.2.4. Matricellular Proteins

These proteins are nonstructural components of the ECM, in contrast to structural elements such as collagens and fibronectin. As their names indicate, they form a bridge between the matrix (matri-) and the cells (-cellular). They bind to other ECM proteins and cell surface receptors, growth factors, cytokines, and MMPs, thereby modulating the activity and accessibility of these factors and mediating enzymatic activities to regulate ECM homeostasis [165]. The main members of this family are thrombospondins, tenascins, osteopontin (also known as SPP1), and CCNs (named from the first three proteins identified in this family: cysteine-rich angiogenic inducer 61 (CYR61), CTGF, and nephroblastoma overexpressed protein (NOV)). Notably, CCN2 is also known as the connective tissue growth factor (CTGF) [166]. Increased serum levels of CYR61 or CCN1 were proposed as biomarkers of HCV-induced HCC post-SVR [14] (see Section 1). The link between HCV pathogenesis and thrombospondin-1 has already been described in Section 3.2.2.1.

##### 3.2.4.1. Tenascins

There are four tenascins, C, R, W, and X, but only tenascins-C and -X are ubiquitously expressed. Tenascin-C is present in all organs during fetal development but weakly expressed in normal adult tissue. During mechanical stress or pathological tissue remodeling, such as wound healing, fibrogenesis, or tumorigenesis, its expression is enhanced. A chronic liver injury leads to the activation of HSCs, with concomitant enhanced secretion of tenascin-C in chronic hepatitis C [104]. Serum tenascin-C has therefore been suggested as a biomarker to discriminate between fibrotic/cirrhotic patients with active hepatitis C from healthy controls and those with HCV eradication after antiviral therapy [105]. Tenascin-C serum levels were found to be good indicators of ongoing hepatic injury and inflammation in fibrotic/cirrhotic patients. After SVR, serum levels of this protein returned to the baseline observed in healthy individuals, suggesting the reversion of HSCs to their quiescent state [105]. More specifically, serum levels of large splice variants of tenascin-C were proposed as useful markers of the inflammatory activity during chronic hepatitis C and in particular of the degree of piecemeal necrosis [167]. However, somewhat discordant results were reported, as tenascin was found underexpressed in the liver ECM of chronically HCV-infected patients, in correlation with the fibrosis stage [59]. In fine, no mechanistic hypothesis was put forward concerning the link between HCV infection, liver disease, and tenascin-C expression.

##### 3.2.4.2. Osteopontin

Osteopontin or secreted phosphoprotein 1 (SPP1) is an adhesive phosphorylated acidic glycoprotein, existing in a full-length form and as cleaved fragments. It is produced by HSCs, cholangiocytes, hepatocytes, HPCs [168], macrophages, and T helper lymphocytes, and its production is enhanced by PDGF and inflammation. Its serum levels are viewed as markers of liver fibrosis from several etiologies (including chronic hepatitis C), significantly correlating with the fibrosis stage, liver insufficiency, portal hypertension, and the presence of HCC [160,169,170,171]. In patients with chronic hepatitis C, osteopontin gene expression enhancement was part of a gene expression signature of moderate (F2) compared to mild (F1) fibrosis [82]. Mechanistic studies revealed a vicious loop linking osteopontin to HCV replication and pathogenesis: endogenous or exogenous osteopontin favors viral replication in hepatocytes [106], as well as assembly and infectivity of viral particles [64]. It likely does so through direct interactions with viral structural and nonstructural proteins (core, NS3, NS5A, NS5B) in the ER and at the membrane of lipid droplets, both sites of HCV replication and assembly. Osteopontin is cleaved intracellularly, and released fragments are secreted in the extracellular space, where they bind CD44 and the αVβ3 integrin in an autocrine and paracrine manner [64]. These cues stimulate HCV replication in the ER through the activation of the focal adhesion kinase (FAK) and promote HCV assembly at lipid droplets. In turn, HCV indirectly stimulates the endogenous production of osteopontin, notably through the activation of oxidative stress [172] and of cellular kinases (MAPK, JNK, PI3-K, MEK1/2). Therefore, a vicious circle is generated. Further investigations revealed that osteopontin enhanced HCV replication in a peculiar population of hepatic cells, i.e., CD44^+^ cancer stem cells [65]. Interestingly, osteopontin was described as a regulator of stemness of these cells, which are among the targets of HCV infection in the liver, and the cells of origin of liver cancers (HCC or intrahepatic cholangiocarcinoma) [173]. This can be linked to the observation that increased serum levels of osteopontin unfavorably correlated with the early recurrence of HCV-related HCC [174]. Osteopontin was also described as an inducer of ductular reactions [172]. In a recent study of ductular reactions in chronic liver disease of various etiologies [47], HPCs (stem cells) from ductular reactions of patients with chronic and active hepatitis C displayed gene signatures related to metabolism and hepatocytes, with gene networks enriched for cell movement and receptor activity not observed in patients with cholangitis. Osteopontin is therefore at a pivotal position: as a regulator of stemness of cells targeted by HCV during infection in relation to its activity as an inducer of ductular reactions and as an enhancer of infection. Along similar lines, HPCs play a key role in HCV pathogenesis, as they are HCV target cells and cells of origin of liver cancers. A more specific link between osteopontin, stemness, ductular reactions, and fibrosis occurrence was established [172]: *osteopontin*^-/-^ transgenic mice less readily developed chemically induced liver fibrosis than wild-type animals, suggesting that osteopontin expression is increased after liver injury. Osteopontin of the diseased liver ECM then decreased hepatocyte proliferation, induced stem cell expansion and ductular reactions, and concomitantly, fueled the upregulation of collagen I in HSCs via its binding to αVβ3 and CD44, contributing to an enhanced fibrogenic response [107,172] together with the modulation of TGF-β signaling [168]. Another vicious circle is therefore unraveled: osteopontin is directly involved in the initial response to the hepatic insult and promotes ductular reactions, which contribute to sustained injury and to liver fibrosis.

Lastly, a genetic link between osteopontin and chronic hepatitis C was discovered; indeed, studies of genetic polymorphism revealed SNPs in the promoter region of osteopontin in chronic hepatitis C patients. In particular, the SNP at nucleotide -443 (C or T) showed an association with the activity of hepatitis C. This activity was defined as the serum levels of alanine aminotransferase (ALT): low (ALT > 30 IU/L), medium (30 < ALT < 80 IU/L), or high (ALT > 80 IU/L). The frequency of T/T homozygotes prevailed in the medium- and high-activity groups, whereas C/T heterozygotes prevailed in the low-activity group [175].

##### 3.2.4.3. CCN2 or CTGF

CCN2 or CTGF is a mitogen and a matricellular protein expressed by hepatocytes, HSCs, vascular endothelial cells, and cholangiocytes [96,176] and overexpressed in chronic liver diseases [177]. CTGF binds to sulfated GAGs and HSPGs (Figure 1), thereby acting as a local reservoir that can attract competent cells to fibrotic sites. It also plays an autocrine role in HSCs, leading to abundant production of ECM molecules. Elevated serum and liver levels of this cytokine were found in fibrotic/cirrhotic patients chronically infected with HCV [177,178]. However, CTGF levels did not correlate with the stage of fibrosis and were higher in patients with progressive fibrosis than in those with end-stage cirrhotic liver disease [176]. HCV induced CTGF overexpression, which in turn stimulated the production of procollagen-I by HSCs, in a TGF-β1-dependent manner [57,58,179]. The viral protein core was suggested as the main trigger of CTGF overproduction [58].

#### 3.2.5. Adhesive Glycoproteins

The main components of this family of ECM proteins are fibronectin, laminins, and nidogens. They are formed of small sugar chains anchored to long polypeptide chains and organized as structural modules, enabling a great diversity of protein–protein and ECM–cell interactions. This modular organization contributes to ECM cohesion. Fibronectin is the main component of the space of Disse in normal liver; it is produced at high rates during liver fibrosis. Fibronectin is found in the plasma as its full-length form produced by hepatocytes and as isoforms produced by activated HSCs, defined by the presence of alternatively spliced domains (fibronectin containing extradomains A or B (EDA or EDB), variable domain). This variable domain is also found glycosylated at a specific threonine residue, known as the oncofetal fibronectin (oFN) isoform [108]. Laminins and nidogens are the main components of basement membranes, involved in the formation of a dense network of ECM proteins. Under physiological conditions, laminins are absent from the space of Disse but present in the basement membrane that surrounds bile ducts and are in contact with HPCs [180].

##### 3.2.5.1. Fibronectin

Circulating levels of the fibronectin isoforms were analyzed in patients with liver fibrosis related to chronic hepatitis C or to other etiologies [108]. Total fibronectin did not correlate with any parameter in either group. However, levels of fibronectin isoforms taken individually were significantly higher in patients with chronic hepatitis C compared to healthy controls, and high levels of EDA and oFN correlated with high scores of liver fibrosis (F2 to F4). Additionally, low levels of both isoforms were associated with the absence of significant liver fibrosis. By contrast, in patients with liver disease unrelated to HCV, none of the isoforms correlated with any parameter. When examining specific aspects of the fibrosis, it was found that EDA elevation significantly correlated with necrosis, and oFN predicted inflammation [181]. A slightly better correlation was found when combining oFN plasma levels with those of some elements of the complement system. Similarly, the combination of oFN with a fraction of the complement correlated better with the fibrosis score than oFN alone [181].

##### 3.2.5.2. Laminin and Nidogen

As described above, HPCs produce during fibrosis an excess of fibrogenic mediators (TGF-β1 and -β2, PDGF, CTGF), which fuel HSC proliferation and activation. In an attempt to understand how the niche of HPCs influences their behavior, Lorenzini et al. analyzed liver tissue from various liver injury models and compared them with healthy tissue and addressed the functional significance of the laminin—HPC interaction. In liver tissue from patients with chronic hepatitis C, close contact between HPC, myofibroblasts, and laminin was observed [182]. Laminin, produced by myofibroblasts and HPC, deposited as a sheath surrounding HPC, which contributed to the cohesion of the niche around HPC. Laminin also deposited in the space of Disse is a phenomenon called the capillarization of sinusoids observed in liver disease related to chronic hepatitis C but not to other etiologies [183]. In vitro experiments revealed that HPCs grown on laminin-enriched ECM maintained their stemness properties, while HPCs grown on fibronectin differentiated into hepatocytes. Laminin strongly repressed the expression of *C/EBPα*, an early hepatocyte gene encoding a liver-selective transcription factor [182]. Laminin therefore contributed to maintaining HPCs in their undifferentiated status, which could fuel the deregulation of HPC proliferation underlying liver cancer.

Nidogen was found expressed on cell membranes of biliary epithelia and not on hepatocytes in normal liver tissue. In HCV-infected diseased tissue, nidogen staining was seen on cell membranes of periportal hepatocytes [183]. However, no functional analysis was performed, which could explain such location.

#### 3.2.6. Elastin

Elastin is a minor ECM component in normal liver, which nevertheless plays a key role to confer strength and elasticity to the organ. It is cross-linked from tropoelastin by LOX to give rise to insoluble elastin fibers, in a similar reaction as collagen fibrillogenesis [24,27]. Elastin fibers are stable components of the ECM, although proteolytically processed by MMP-2, -9, and the elastase MMP-12. TGF-β1-mediated activation of HSCs leads to increased expression of tropoelastin [184], and the deposition of insoluble elastic fibers in the diseased liver could lead to the irreversibility of fibrosis [27]. Elastin was found overexpressed in fibrotic and cirrhotic livers compared to normal organs. This overexpression correlated with the severity of the hepatic disease [102]. In liver biopsies from patients with chronic hepatitis C, increasing amounts of elastin correlated with the severity of liver fibrosis [83,84]. This was accompanied by an increase of expression of fibulin-5 and microfibrillar-associated protein-4, proteins associated with elastic fibers and involved in elastogenesis [59,185,186]. More selectively, microfibrillar-associated protein-4 was found overexpressed in liver biopsies [84], more specifically in the ECM fraction of liver biopsies [59] and also at high levels in the serum of HCV-positive patients [84], correlating with the severity of the disease [109,110]. Mechanistically, elastin processed into small peptide fragments acts as a chemoattractant for cells of the immune system that will locally secrete inflammatory cytokines (Figure 1). Elastin peptides can also stimulate the proliferation of activated HSCs and their secretion of MMP-12 [27], thereby aggravating the imbalance between MMP and TIMP and contributing to fibrogenesis (Figure 2).

#### 3.2.7. TGF-β and HCV Pathogenesis

In most organs, such as the liver, TGF-βs are secreted as a large latent TGF-β complex, formed by the mature TGF-β protein noncovalently attached to the precursor protein (latency-associated peptide), linked to large latent TGF-β-binding proteins (LTBPs) [187]. LTBPs play a key role in the activation of this latent complex by targeting it to the cell surface followed by its secretion into extracellular areas, where activation likely occurs, notably via thrombospondin-1 (see [61], Section 3.2.2) and furin [72,188]. Of the three TGF-β isoforms (TGF-β1, -β2, and -β3) described in mammals, TGF-β1 is the most extensively studied in human liver diseases. Under normal conditions, TGF-β1 is mainly secreted from Kupffer cells, while hepatocytes only secrete small amounts [72]. Upon any liver injury in general, and chronic hepatitis C in particular, high levels of TGF-β1 and -β2 are secreted in the space of Disse by hepatocytes and neighboring cells [57,66], fueling fibrogenesis [34,189] (Figure 2), whereas TGF-β3 was reported as an antifibrotic cytokine in the liver. Indeed, TGF-β3 was shown to inhibit TGF-β1 expression at the transcriptional level and suppress collagen synthesis [190]. TGF-β1 and -β2 secretion contributes to HSC activation and transformation into myofibroblast-like cells [66]. Indeed, TGF-β1-mediated activation of HSCs [191] results in: (i) vitamin A loss accompanied by a decrease in serum retinoid levels [192]; (ii) α-SMA expression [42,58,61]; (iii) greater TGF-β1 secretion [72] in a vicious loop. TGF-βs are therefore key regulators of fibrosis, involved in chronic liver diseases, which contribute to all disease stages, from initial liver injury to fibrosis, cirrhosis, and HCC [193].

In patients with chronic hepatitis C, increased serum levels of TGF-β1 and -β2 were reported [67,87], together with high amounts of TGF-β1 and -β2 mRNA in liver specimens [194], compared to healthy individuals. These correlated with the degree of liver fibrogenesis but not with HCV RNA levels [67,111,195]. Higher circulating levels were also found in patients with HCC, compared to patients with liver cirrhosis, and these levels were the highest when the liver disease was caused by HCV infection or HBV/HCV coinfection, compared to other etiologies [113]. TGF-β1 mRNA in liver biopsies strongly correlated with procollagen type I mRNA as a further indication of the link between TGF-β1 expression and liver fibrosis/cirrhosis [111]. LTBP-1 and TGF-β1 protein levels were found upregulated in liver tissue from patients with chronic hepatitis C [112,195]. Analyses selectively performed on the ECM fraction of liver biopsies revealed large deposits of LTBP-1 and -4 in HCV patients at stage F3, suggestive of a pivotal role of the large latent TGF-β complex at the F2–F3 transition [59]. The mechanistic crosstalk between HCV and TGF-β synthesis and activity has been investigated in various in vitro or in vivo models of chronic HCV infection. HCV-infected hepatocytes implanted into mice generated nodular tumors enriched in cancer stem-like cells. These hepatocytes were shown to recruit activated murine fibroblasts into the xenograft stroma by secretion of TGF-β1 [194]. This led to stromal changes, such as activation of HSCs (α-SMA expression and enhanced synthesis of MMP-2) and the appearance of markers of tumor-associated fibroblasts: collagen I, CTGF, vimentin, and fibroblast-specific protein 1. HCV infection also resulted in the overproduction of TGF-β1 in liver cells isolated from patients with chronic hepatitis C [66]. HCV core, expressed in human hepatoma cells, strongly enhanced TGF-β1 mRNA expression in these cells and upregulated the TGF-β1 promoter [196]. The MAPK pathway was found involved in the engagement of TGF-β1 by the HCV core protein. Other studies have similarly addressed the crosstalk between this viral protein and TGF-β1-mediated fibrogenesis, as described earlier in relation to enzymes of the ECM (Section 3.2.2.1 and Section 3.2.2.2). The expression of HCV core and E1 and E2 proteins in human hepatocytes revealed that core and E2 were responsible for the enhanced secretion of TGF-β1 through the overproduction of the glucose-regulated protein 94 (GRP94), a chaperone protein of the ER lumen and an ER stress marker [66]. This further emphasizes the link between TGF-β1 production and oxidative and ER stress, all induced by HCV [72,77]. GRP94 upregulation would engage the NF-κB pathway, which would, in turn, trigger the enhancement of TGF-β1 production. The exogenous addition of the HCV NS3 protease to cultured hepatocytes led to increased expression of collagen I, TGF-β1, and TGF-β type I receptor [73]; additionally, NS3 directly interacted with the TGF-β type I receptor at the surface of HCV-infected cells and in HCV-infected chimeric mice, an anti-NS3 antibody attenuated HCV-induced liver fibrosis. HCV NS3 present in extracellular areas, possibly arising from the leakage from injured hepatocytes, would therefore function via its direct binding to the TGF-β type I receptor and its activation, thereby enhancing liver fibrosis [73]. Moreover, the functionally active protease domain of NS3 was found required for TGF-β1 activity, i.e., for the activation of the TGF-β1 promoter [72]. This implies the presence of the cofactor NS4A with NS3. NS3-4A could then enhance the proteolytic activity of thrombospondin-1 and furin for the cleavage of the latent TGF-β complex and subsequent activation of TGF-β. NS3-4A also interacted with SMURF2, a negative regulator of TGF-β signaling. Through this interaction, NS3-4A blocked the negative regulation of TGF-β signaling, thereby enhancing the cellular response to TGF-β [75]. Accordingly, hepatocytes expressing either nonstructural proteins (NS3 to NS5B) or NS3-4A of HCV, once stimulated by TGF-β, exhibited a protumoral transcriptional program, with enhanced expression of genes involved in cell proliferation, negative regulation of cell differentiation, epithelial-to-mesenchymal transition, and vasculature development [75], all genes being related to carcinogenesis. Expressing HCV NS5A into hepatocytes also induced the production of high levels of TGF-β1, and NS5A was found important for the activation of the TGF-β1 promoter [72]. Interestingly, NS5A associates with the membrane of the ER [197], where it contributes to ER stress and induces an ER-to-nucleus signal transduction pathway involving NF-κB [77,198]. This pathway could contribute to chronic inflammation and liver fibrosis. Again, this confirms the link between HCV infection, virus-induced ER stress, and TGF-β1 overexpression and activity. As already noted for NS3, NS5A directly interacted with the TGF-β type I receptor at the surface of cells expressing NS5A [78], and together with other viral proteins, might therefore contribute to liver fibrogenesis through the engagement of TGF-β signaling.

TGF-β2 was found upregulated by the coexpression of two proteins of HCV, core and NS2, most markedly in hepatocytes compared to HSCs and Kupffer cells [67]. Mechanistically, HCV-induced ER stress following hepatocyte infection caused the translocation of the liver-enriched and hepatocyte-specific transcriptional regulator cAMP-responsive element-binding protein H (CREBH) from the ER to the Golgi apparatus and its subsequent activation. The activated CREBH was then transported to the nucleus, where it targeted and bound the DNA CREBH response element and the CRE responsive element-like sequence. Both elements modulate the expression of their target gene, *TGFβ2* [190]. Through this pathway, HCV therefore enhanced TGF-β2 expression in hepatocytes [67]. After its release in the extracellular milieu, TGF-β2 exerted effects on hepatocytes and HSCs in autocrine and paracrine manners, increasing the expression of profibrotic molecules (TGF-β1 and -β2, α-SMA, collagen I), thereby promoting liver fibrosis [67].

Endoglin is a transmembrane glycoprotein belonging to the TGF-β receptors family, where it constitutes, together with betaglycan, the TGF-β type Ⅲ receptor family. Endoglin interacts with the TGF-β receptors types I and II and acts as a key switch by producing different variant forms, adjusting ligand affinity, and creating links with a versatile receptor network, thereby modulating the specific outcome of TGF-β-dependent and -independent pathways [199]. It is expressed as a dimer at the surface of proliferating vascular endothelial cells of quiescent and activated HSCs but not of hepatocytes. It binds TGF-β1 and -β2, and in HSCs, it is associated with the TGF-β type Ⅱ receptor and shows the highest expression during maximal cell activation. It is activated by phosphorylation through the activity of the TGF-β type Ⅱ receptor part. Its expression is upregulated during liver damage and transiently induced in HSC by TGF-β1. Circulating levels of endoglin were higher in patients with chronic hepatitis C than in healthy individuals [100] and correlated with the severity of the liver disease, with the highest values in HCV-positive cirrhotic patients. Intra-hepatic levels of endoglin were also higher in HCV-infected livers than in uninfected biopsies [68,100], as also observed for TGF-β1 levels. Endoglin expression in the liver correlated with the METAVIR score. This suggests that increased levels of released TGF-β1, linked to HCV-induced liver fibrosis/cirrhosis, may be responsible for the endoglin upregulation in a positive feedback loop where the ligand (TGF-β1) stimulates the expression of (one of) its receptor (endoglin). This could possibly create a vicious circle, with sustained or continuous activation of the TGF-β receptors, leading to the production of overly abundant components of the ECM by activated HSCs and therefore to fibrosis. This poses endoglin as a key element of HCV pathogenesis, playing a crucial role in liver fibrogenesis. Mechanistic studies revealed that endoglin upregulation in HCV-infected hepatocytes was mainly due to the HCV core protein [68]. In these cells, core-induced overexpression of endoglin contributed to the activation of the TGF-β signaling pathway, with the transcription of target genes involved in cell proliferation and acquisition of (cancer) stem cell properties. In an attempt to discover novel determinants of HCV-related liver fibrosis progression, a joint French/Swiss study group identified an endoglin variant, Thr5Met, the frequency of which was higher among HCV/fibrosis patients than among HCV/controls [200]. This variant was even depleted in patients without fibrosis. An additional SNP was found in a regulatory region of the endoglin gene in relation to increased risk of liver fibrosis in HCV patients and predicted to decrease the DNA-binding affinity of HNF4α (involved in the expression of liver-specific genes) [200]. This would lead to a lower transcription of liver-specific genes, and, as mentioned earlier, to the maintenance of hepatic progenitors in their undifferentiated state, leading to the occurrence of (HCV-infected) cancer stem cells underlying HCC [47].

## 4. Are HSCs Direct Targets of HCV Infection?

Studies addressing the capability of HCV to directly interact with HSCs, that could contribute to a direct fibrogenic effect, have reported conflicting data. Florimond and coworkers showed that human liver myofibroblasts, isolated from liver specimens, and the immortalized HSC cell line LX-2 [201] were not infectable by HCV due to the lack of receptors essential to its recognition and internalization; they also reported that these cells could not support HCV replication [202]. Conversely, Aoudjehane and coworkers showed that human liver myofibroblasts possessed all key receptors necessary and sufficient for HCV entry and supported HCV infection [203]. Infection was followed by overexpression of α-SMA and collagens I and IV. These conflicting data might result from the use of different cell lines and of human liver myofibroblasts infected at different passages. Interestingly, activated HSCs expressed mRNA of all receptors required for productive HCV infection, and incubation of these cells with soluble HCV core or protease NS3 led to the activation of oxidative stress and the stimulation of NF-κB-dependent gene expression pathways [62]. Core preferentially activated pathways involved in cell proliferation, while NS3 acted as a trigger for proinflammatory pathways. The expression of core or NS3 into HSCs led to the increased production of α-SMA, procollagen-I, and TGF-β1 [62]. Although these data did not demonstrate that HSCs could support HCV replication, they suggested that they were potential targets of HCV infection. A recent concept emerged that exosomes secreted by HCV-infected hepatocytes might be “agents” of communication between these cells and HSCs. Indeed, HSCs were found able to internalize exosomes from infected hepatocytes, which led to the upregulation of profibrogenic components (α-SMA, collagens I and III, TIMP-1, MMP-2, CTGF, and TGF-β1) [204]. This upregulation was triggered by the micro-RNA miR-19a, shuttled between infected hepatocytes and HSCs by exosomes. MiR-19a targeted the SOCS3/STAT3 axis, ultimately leading to the engagement and activation of the profibrotic TGF-β1 signaling pathway. This study therefore unraveled a direct mechanism of HSC activation by HCV-infected hepatocytes, although not based upon the infection of HSCs [204].

However, additional studies are needed to fully settle this question, and one must keep in mind that human liver myofibroblasts correspond to activated HSCs, which leaves open the question of HCV infection of quiescent HSCs [205]. Nevertheless, the possibility that (quiescent or activated) HSCs might be directly modulated by the virus during liver invasion enlarges our mechanistic perspectives on the progression of liver disease during chronic infection.

## 5. Fibrosis Reversal in the Era of DAAs in HCV-Induced Liver Fibrosis

The main angle of attack of hepatitis C-related liver fibrosis is to reduce or eradicate the primary disease, i.e., curing viral infection. With high SVR rates achieved, most patients treated with DAAs have seen their clinical symptoms regress, in particular fibrosis. The recent development of transient elastography has placed the measured liver stiffness as an accurate surrogate index of liver fibrosis, in particular to evaluate the efficacy of DAA treatment [206]. However, such treatment does not fully restore the altered cytokine and chemokine milieu [12], and patients at advanced stages of disease may remain at risk of liver complications. Combining DAAs with other antifibrotic strategies may be desirable, such as therapies aiming at the stimulation of matrix degradation, in particular the inhibition of LOXL2. The safety, tolerability, and potential efficacy of the anti-LOXL2 monoclonal antibody simtuzumab have been recently assessed in a study of three cohorts of patients: chronically infected by HCV, infected by the human immunodeficiency virus (HIV), or coinfected by HCV and HIV (ClinicalTrials.gov Identifier: NCT01707472 and [207]). Although the treatment was well tolerated, no clinical benefit was observed, with no significant changes in fibrosis score before and after therapy. This might be due to the poor accessibility of simtuzumab to the site of fibrosis, linked to the collagenous consistency of the connective tissue. Other strategies under examination in animals might raise hopes of novel therapies aimed at fibrosis regression, in addition to SVR in chronic hepatitis C patients (reviewed in [17]). Some strategies target intracellular signaling to restore it to normal, such as inhibitors of tyrosine kinase receptors, which are signal transducers of several cytokines. Others aim at inhibiting fibrogenesis by interfering with its main actors, TGF-β1 and CTGF; inhibitors of the TGF-β1 pathway could either block circulating cytokine, antagonize its receptors, and/or block its activation at the cell surface. The monoclonal antibody against CTGF, FG-3019, is currently under clinical investigation in lung fibrosis for safety and tolerability (ClinicalTrials.gov Identifier: NCT01890265) and might be applied to liver fibrosis. Other strategies consist of increasing the apoptosis of activated HSCs through the inhibition of antiapoptotic proteins or transcription factors such as NF-kB. However, although several strategies have been tested in clinical trials lately, their antifibrotic effects have been limited or absent. Thus, to date, no approved therapy exists for liver fibrosis [208].

## 6. Conclusions and Perspectives

In the era of DAAs, which raises hopes of eradicating HCV, HCV infection remains a leading cause of hepatic failure due to advanced liver disease and HCC because curing the infection does not fully restore liver homeostasis. Furthermore, DAA treatment alone may not be sufficient for a complete cure of fibrosis, as several factors other than the virus contribute to liver deterioration. Lastly, patients under antiviral therapy variably respond to the regression of fibrosis. The mechanism of HCV-induced liver disease is a multifaceted process, as various host genes are altered, and host cells respond to infection/viral components by mobilizing or producing enzymes, growth factors, and chemokines, which activate quiescent HSCs. HCV chronic infection leads to a deep remodeling of the entire liver ECM architecture through direct interactions between viral, ECM, and cellular proteins and indirect effects (e.g., promotion of oxidative and ER stress, inflammation, and stemness). HCV-induced overexpression of TGF-β, the most potent profibrogenic cytokine, contributes to HCV replication and to the activation of HSCs, the promotion of their survival, and the inhibition of HSC apoptosis, mechanisms by which liver disease progresses. Consequently, several general mechanisms involved in liver fibrosis/cirrhosis development contribute to tumorigenesis. TGF-β signaling facilitates HCV replication in hepatocytes and could promote the survival of precancerous cells; furthermore, HCV replicates at higher rates in liver cancer stem cells.

Thus, efforts toward a deeper comprehension of host/virus/ECM interactions and of the underlying mechanisms by which hepatic dysfunctions emerge, spread, and persist after HCV infection are therefore still needed in order to develop therapies that cure liver disease in addition to curing infection.

## Figures and Tables

**Table 1:** Main differences between HBV and HCV pathogenesis.

Virus	HBV	HCV
Viral family	*Hepadnaviridae*	*Flaviviridae*
Genome	DNA and cccDNA	RNA
Life cycle	Genome integration, expression of HBx protein, insertional activation of cellular oncogenes, cccDNA (minichromosome)	Exclusively cytoplasmic
Persistence	Nucleus-located cccDNA	Chronic inflammation, oxidative stress, alterations in cellular signaling and metabolism

**Table 2:** Proteins of HCV reported being related to proteins of the ECM or cytokines. *, Direct interaction with the indicated HCV protein; ∞, modulation of expression; ◊, modulation of signaling.

HCV Proteins	ECM Proteins or Cytokines
Capsid core	LOX ∞ [61] Procollagen I ∞ [62] Collagen I ∞ [61] MMP-2 ∞ [58]
	MMP-9 ∞ [63]
	COX-2 ∞ [63]
	Syndecan-1 * [31]
	Thrombospondin-1 ∞ [61]
	Osteopontin * [64,65]
	CTGF ∞ [58]
	TGF-β1 ◊ [58,61,62,66]
	TGF-β2 ◊ [67]
	Endoglin ∞ [68]
Envelope glycoproteins E1 and/or E2	Glypican-3 * [69]
	TGF-β1 ◊ [66]
Cysteine autoprotease NS2	MICA ∞ [70] TGF-β2 ◊ [67]
Serine protease and helicase NS3	Procollagen I ∞ [62] MMP-9 ∞ [71]
	COX-2 ∞ [71]
	Thrombospondin-1 [72]
	Osteopontin * [64]
	TGF-β1 ◊ [62,72]
	TGF-β type I receptor * [73]
NS3 with its cofactor NS4A	MMP-9 ∞ [71]
	COX-2 ∞ [71] MICA ∞ [74]
	TGF-β ◊ [72,75]
NS4B	MMP-2 ∞ [76]
NS5A	MMP-2 ∞ [63]
	MMP-9 ∞ [63]
	COX-2 ∞ [63]
	Thrombospondin-1 ∞ [72]
	Osteopontin * [64]
	TGF-β1 ◊ [72,77,78]
RNA-dependent RNA polymerase NS5B	Osteopontin * [64]
	MICA ∞ [70] TGF-β ◊ [75]

**Table 3:** Upregulation or downregulation of indicated ECM proteins or cytokines in connection with METAVIR liver fibrosis stages or HCC in chronically HCV-infected patients ^a^.

ECM Proteins/Cytokine	F0/F1	F2	F3	F4	HCC	References
Collagens I, III, V	F1					[45,59,60,79,80,81,82,83]
Collagen XII						[59,84]
Collagen XIV						[59,84]
Collagen XVI						[59]
Collagen XVIII						[59]
PIIINP	F1					[85,86,87]
MMP-2, -7, -9	F1					[63,82,88,89,90]
TIMP-1						[82,86,88,91,92]
ADAM-TS1						[93]
ADAM-TS2						[94]
Xylosyltransferase-2	F1					[95,96]
Glypican-3						[97,98,99]
Hyaluronic acid						[87,100,101]
Decorin	F1					[92]
Biglycan						[59]
Fibromodulin						[60]
Lumican						[59,81,84,102]
Versican	F1					[93,103]
Tenascin-C						[104,105]
Osteopontin	F1					[82,106,107]
Fibronectin						[103,108]
Fibronectin isoforms						[108]
Elastin						[59,83,84,102]
MFAP-4 ^†^	F1					[84,109,110]
Fibulin-5						[84]
TGF-β1 (protein, mRNA)						[59,100,103,111,112]
TGF-β1 (serum levels)	F1					[87,113]
TGF-β2	F1			F0		[67]
Endoglin (protein, serum levels)						[100]
Endoglin (mRNA) ^§^						[68]

^a^ Color codes: green, upregulation; dark green: higher upregulation; blue, downregulation; dark blue: higher downregulation; grey, no change; magenta, no correlation with liver fibrosis stage. ^†^ MFAP-4, microfibrillar-associated protein-4 (associated with elastin fibers). ^§^ Endoglin mRNA was found upregulated in chronically HCV-infected patients compared to noninfected patients but not correlating with liver fibrosis stage.

## Data Availability

Data sharing not applicable: no new data were created or analyzed in this study. Data sharing is not applicable to this article.

## Abbreviations

ADAM	a disintegrin and metalloprotease
ADAM-TS	ADAM with thrombospondin motifs
ALT	alanine amino-transferase
COX-2	cyclo-oxygenase-2
CREBH	cAMP-responsive element binding protein H
CS	chondroitin sulfate
CTGF	connective tissue growth factor
DAAs	direct-acting antivirals
ECM	extracellular matrix
ER	endoplasmic reticulum
GAG(s)	glycosaminoglycan(s)
GPC3	glypican-3
HA	Hyaluronan or hyaluronic acid
HBV	hepatitis B virus
HCC	hepatocellular carcinoma
HCV	hepatitis C virus
HGF	hepatocyte growth factor
HNF4α	hepatocyte nuclear factor 4α
HPC	hepatic progenitor cells
HSC(s)	hepatic stellate cell(s)
HSPG(s)	heparan sulfate proteoglycan(s)
IP-10	interferon gamma-inducible protein 10
LOX	lysyl oxidase
LOXL	LOX-like
LTBP	large latent TGF-β-binding protein
MMP(s)	matrix metalloprotease(s)
NAFLD	nonalcoholic fatty liver disease
NF-κB	nuclear factor-κB
NK	natural killer cell
PDGF	platelet-derived growth factor
PG(s)	proteoglycan(s)
SMA	smooth muscle actin
SNP	single-nucleotide polymorphism
SVR	sustained virological response
TGF-β1 and -β2	transforming growth factor-β1 and -β2
THBS1	thrombospondin-1 gene
TIMP	tissue inhibitor of MMP
TLR	toll-like receptor
